# Implementation of an innovative web-based conference table for community-dwelling frail older people, their informal caregivers and professionals: a process evaluation

**DOI:** 10.1186/1472-6963-12-251

**Published:** 2012-08-15

**Authors:** Sarah HM Robben, Marieke Perry, Mirjam Huisjes, Leontien van Nieuwenhuijzen, Henk J Schers, Chris van Weel, Marcel GM Olde Rikkert, Theo van Achterberg, Maud M Heinen, René JF Melis

**Affiliations:** 1 Department of Geriatric Medicine, 925, Radboud University Nijmegen Medical Centre, P.O. Box 9101,, 6500 HB, Nijmegen, The Netherlands; 2 Department of Primary and Community Care, Centre for Family Medicine, Geriatric Care and Public Health, 117, Radboud University Nijmegen Medical Centre, P.O. Box 9101, , 6500 HB, Nijmegen, The Netherlands; 3 Scientific Institute for Quality of Healthcare, 114, Radboud University Nijmegen Medical Centre, P.O. Box 9101, , 6500 HB, Nijmegen, The Netherlands

**Keywords:** E-health, Implementation, Process evaluation, Frail older people, Primary care

## Abstract

**Background:**

Due to fragmentation of care, continuity of care is often limited in the care provided to frail older people. Further, frail older people are not always enabled to become involved in their own care. Therefore, we developed the Health and Welfare Information Portal (ZWIP), a shared Electronic Health Record combined with a communication tool for community-dwelling frail older people and primary care professionals. This article describes the process evaluation of its implementation, and aims to establish (1) the outcomes of the implementation process, (2) which implementation strategies and barriers and facilitators contributed to these outcomes, and (3) how its future implementation could be improved.

**Methods:**

Mixed methods study, consisting of (1) a survey among professionals (n = 118) and monitoring the use of the ZWIP by frail older people and professionals, followed by (2) semi-structured interviews with purposively selected professionals (n = 12).

**Results:**

290 frail older people and 169 professionals participated in the ZWIP. At the end of the implementation period, 55% of frail older people and informal caregivers, and 84% of professionals had logged on to their ZWIP at least once. For professionals, the exposure to the implementation strategies was generally as planned, they considered the interprofessional educational program and the helpdesk very important strategies. However, frail older people’s exposure to the implementation strategies was less than intended. Facilitators for the ZWIP were the perceived need to enhance interprofessional collaboration and the ZWIP application being user-friendly. Barriers included the low computer-literacy of frail older people, a preference for personal communication and limited use of the ZWIP by other professionals and frail older people. Interviewees recommended using the ZWIP for other target populations as well and adding further strategies that may help frail older people to feel more comfortable with computers and the ZWIP.

**Conclusions:**

This study describes the implementation process of an innovative e-health intervention for community-dwelling frail older people, informal caregivers and primary care professionals. As e-health is an important medium for overcoming fragmentation of healthcare and facilitating patient involvement, but its adoption in everyday practice remains a challenge, the positive results of this implementation are promising.

## Background

Our current healthcare system is not well equipped to meet the needs of the growing population of frail older people [1]. Due to fragmentation, care for a single frail older person is often provided by a number of professionals who work for a variety of organizations and services, which results in discontinuity of care [2,3]. Also, the healthcare system is not designed to facilitate the incorporation of patient perspectives in decision making, which becomes even more difficult when care is delivered by an interprofessional team [4,5]. This is unfortunate, as involving patients in their care is mandatory and can improve patient outcomes [6,7].

Information Technology has the potential to diminish these problems, by means of a multidisciplinary shared Electronic Health Record that is accessible to patients as well [1,8]. Therefore, we developed a program which included such a record: the Health and Welfare Information Portal (ZWIP). The program aimed to facilitate shared decision-making and self-management by frail older people and informal caregivers, as well as to reduce fragmentation of care by improving collaboration among professionals involved.

As the program consisted of a number of interacting components and was delivered to several different general practices, conducting a process evaluation to study its implementation was considered critical [9]. First, because information concerning barriers and facilitators experienced during the implementation may be able to guide improvements to the implementation plan and the intervention itself [10,11]. Second, because it can help identify critical factors for the intervention’s implementation in other settings or by other research groups [11]. Such process evaluations are especially useful for e-health interventions, as the number of studies concerning their implementation is limited [12], while their adoption remains a challenge [13]. Therefore, this article aims to establish (1) the outcomes of the implementation process of the ZWIP, (2) which implementation strategies and experienced barriers and facilitators contributed to these outcomes, and (3) how its future implementation could be improved.

## Methods

We evaluated the implementation of the ZWIP with a mixed methods study, consisting of (1) a quantitative evaluation by means of a survey and data collected during the implementation and use of the ZWIP, followed by (2) a qualitative evaluation by means of semi-structured interviews with purposively selected participants. The local ethics committee, the Committee on Research involving Human Subjects Arnhem-Nijmegen, reviewed the study and stated that no formal approval was required.

### Participants

Participants of the study were community-dwelling frail older people, who were patients of participating general practices in the province of Gelderland or Noord-Brabant, the Netherlands; their informal caregivers; and healthcare and welfare professionals involved in their care. They participated in the ZWIP during its implementation phase, which started in September 2010 and ended on the first of July 2011. We monitored the use of the ZWIP and its implementation strategies for both frail older people and professionals. Further, professionals were surveyed and interviewed. We chose not to survey or interview frail older people and informal caregivers as the project had already been time-consuming for them, and they would be surveyed and interviewed as part of the project’s future effect evaluation as well.

### Intervention: the Health and Welfare Information Portal

The ZWIP was developed by means of Intervention Mapping [14], a method for the systematic development of evidence-informed interventions. Throughout this development, future users, i.e. primary care professionals and geriatricians (n = 15), as well as (frail) older people and informal caregivers (n = 14), were involved extensively trough their participation in working groups. These working groups started with participants specifying which problems related to interprofessional collaboration and self-management by frail older people should be solved by the ZWIP, for example not knowing which professionals are involved in the care of a particular frail older person and professionals not being able to contact each other. Then, theories matching the identified determinants of these problems were used to support the development of the intervention. These included Social Cognitive Theory[15], Goal Setting Theory [16] and elements of organizational change theories [17]. The involvement of the target populations continued during the iterative development process of the ZWIP.

The ZWIP can be considered a combination of an Electronic Health Record accessible to the frail older person, informal caregiver and all professionals involved, with a tool for interprofessional and patient-professional communication. The ZWIP consists of (1) information about the frail older person’s health, functioning and social situation, contact information about professionals involved in their care, and care-related goals formulated by or with the frail older person, (2) a secure messaging system for communication between the frail older person and one or more professionals or between professionals, and (3) tailored educational materials for the frail older person and informal caregiver. The frail older persons hold the key to the ZWIP, as they decide which professionals are granted access to their personal ZWIP. The ZWIP can be entered by logging on to a website which conforms to Dutch security regulations. This website, which runs in Dutch, can be accessed from any computer. Frail older persons and their informal caregivers can log on by means of a shared user name and password, while professionals need a security token for logging on. Additional file 1 presents a movie which demonstrates the use of the ZWIP by a frail older person and her informal caregiver.

### Implementation of the Health and Welfare Information Portal

The ZWIP implementation team consisted of the project manager, physicians, a nurse, a nurse scientist experienced with implementation, and research assistants working for the department of Geriatric Medicine of the Radboud University Nijmegen Medical Centre. They implemented the ZWIP using tailored implementation strategies for each target population, i.e. frail older people and informal caregivers, professionals and the employing organizations of professionals. An overview of the implementation strategies used is provided in Table 1.

**Table 1:** Implementation strategies as planned for each target population

	Frail older people and informal caregivers	Professionals	Employing organizations
Recruitment strategies	Involvement in development	Involvement in development	Financial compensation
	Flyers about the program	Flyers about the program	Educational program for employees
	Involvement of GP who asked for participation in screening	Starting with intrinsically motivated early adopters by inviting general practices affiliated with the university hospital to participate	
	Involvement of informal caregiver		
Supporting strategies	Visit by a volunteer who instructs them in the use of the ZWIP	Interprofessional educational program (Continuing Medical Education credits available)	
	Internet and paper version of ZWIP	E-learning	
	Telephonic helpdesk	Coaching of professionals conducting the screening	
		Telephonic helpdesk	
		Newsletter	
		Tailoring of intervention to meet local circumstances	
		Deviations from inclusion criteria, such as including younger frail older people, allowed to gain experience	
		Financial compensation	
		Incentives such as the general practice receiving a cake after including the first frail older person	

We invited general practices affiliated with this University Hospital or involved in the program’s development to participate in the ZWIP, which was made available to them at no charge for the duration of the study. The participating practices invited local primary care professionals from all relevant disciplines involved in the care of frail older people, such as physiotherapists, district nurses and social workers, to take part in the programs’ interprofessional educational program. This program, for which continuing medical education credits were available, addressed screening for frailty, self-management support, interprofessional collaboration, and use of the ZWIP during three three-hour workshop meetings. In addition, professionals received coaching in specific components of the program, and were supported by a telephonic helpdesk. Further, financial compensation was provided for the time invested in the program.

The general practices screened their populations of ≥70 years for frailty using a two-step screening questionnaire (Easycare-TOS). In the first step, the general practitioner (GP) selected patients which were considered (possibly) frail. In the second step, the thus selected patients were screened for frailty on the physical, psychological and social domain during a home visit by a nurse or gerontological social worker. During a second home visit, all people who were frail were invited to participate in the ZWIP. If they gave informed consent, a ZWIP was installed for them.

We supported frail older people and informal caregivers in using the ZWIP by a number of strategies, such as offering a visit by a volunteer who could demonstrate the ZWIP, having a telephonic helpdesk available, and making the ZWIP available in print when this was preferred.

### Quantitative evaluation

The quantitative evaluation of the implementation of the ZWIP consisted of a survey for professionals, and an evaluation of the data collected about the use of the implementation strategies and the data from the ZWIP itself. Data collected included the numbers of older people screened, the number of participants, the number of messages sent, the number of professionals participating in a frail older person’s ZWIP, the number of participants who logged on to the ZWIP, the number of calls to the telephonic helpdesk and the number of visits to frail older people by volunteers.

The survey for professionals was sent out at the beginning of July 2011. The survey was developed building on existing questionnaires used previously to evaluate the implementation of complex interventions and on previous experience of the authors. The survey included questions concerning demographics, time spent on using the ZWIP, perceived value of the implementation strategies, and barriers and facilitators for the use of the ZWIP. We used separate questionnaires for GPs, for nurses and gerontological social workers conducting the screening, and for other professionals. Participants were asked to fill out the survey online, those who did not respond were sent a paper version.

### Qualitative evaluation

The qualitative evaluation consisted of semi-structured interviews about experiences with the implementation process and perceived barriers and facilitators for the use of the ZWIP. A topic list for these interviews was developed by members of the research group and was adjusted until consensus was reached. We conducted these interviews with 12 purposively selected professionals, who had a variety of experiences with the implementation process of the ZWIP. This was arranged by selecting professionals from several disciplines and with different roles in the implementation process, who came from three general practices with varying levels of adoption of the ZWIP. In addition, we interviewed members of the implementation team, who were not involved in conducting its evaluation. Interviews were conducted by members of the research group (LvN, MP, SR) and were transcribed verbatim by a research assistant.

### Analysis

We used descriptive statistics to describe baseline characteristics of participants, data collected about the implementation and the actual use of the ZWIP, and data derived from the survey for professionals. The qualitative data gathered in the semi-structured interviews were analyzed by two members of the research group (MP, SR) using content analysis [18]. Interviews were conducted in parallel with data analysis, using Atlas.ti to support this. We conducted interviews until theoretical saturation was achieved.

## Results

### Participants

Fourteen general practices were invited to participate in the study, seven of these (50%) agreed to participate. The characteristics of these practices are shown in Table 2. In total, 290 frail older people and 169 professionals participated in the ZWIP.

**Table 2:** Characteristics of participating general practices

	**General practice 1**	**General practice 2**	**General practice 3**	**General practice 4**	**General practice 5**	**General practice 6**	**General practice 7**
Patients ≥ 70 years, %	11.1	9.4	3.0	9.9	7.5	5.7	7.3
Setting	Village	Village	City	City	Village	City	City
Collaboration in MTM and PTAMs	Yes	Yes	Yes	Yes	Yes	Yes	Yes
Use of e-consulting	No	No	Yes	No	Yes	No	No
Start of implementation	September 2010	September 2010	October 2010	November 2010	December 2010	November 2010	January 2011
Professionals filling out the questionnaire, n							
General Practitioners (n = 34)	3	3	7	6	4	8	3
Professionals conducting screening (n = 22)	2	2	5	5	2	3	3
Other professionals (n = 62)^a^	13	8	4	8	6	9	7

MTM = Multidisciplinary Team Meetings; PTAMs = Pharmacotherapy Audit Meetings; ^a^Total n = 62 as general practice is unknown for 7 other professionals.

A total of 158 professionals received a survey, i.e. 39 GPs, 26 nurses and gerontological social workers conducting the screening, and 93 other professionals. Eleven professionals could not be reached. Hundred-eighteen professionals (75%) returned the questionnaire, 34 were GP’s, 22 were nurses and social workers who conducted the screening and connected frail older persons to the ZWIP, and 62 were other professionals, such as physiotherapists, pharmacists, nurses and social workers.

Twelve purposively selected professionals participated in the semi-structured interviews. Three of them were GPs, three were nurses and gerontological social workers conducting the screening at the homes of participants, three were other professionals, i.e. pharmacists (n = 2) or physiotherapist (n = 1) and three were members of the implementation team, i.e. project manager, nurse providing coaching, or research assistant.

### Outcomes of the implementation process

The 290 frail older people who had a ZWIP account installed constituted 49% of all frail older people invited. The percentage of frail older people agreeing to participate varied over the separate general practices. Interviewees suggested that this may have been caused by variation in local professionals’ attitudes towards the ZWIP, as well as by variation in computer literacy due to social-economic differences between the general practices. An overview of the outcomes of the implementation process is provided in Table 3.

**Table 3:** Outcomes of the implementation of the ZWIP

**End of implementation: 1 July, 2011**	**General practice 1**	**General practice 2**	**General practice 3**	**General practice 4**	**General practice 5**	**General practice 6**	**General practice 7**	**Total**
Number of older people screened, n	705	365	284	426	200	621	169	2770
Number of older people screened who were frail, n (%)	71 (10.1)	80 (21.9)	49 (17.3)	116 (27.2)	25 (12.5)	213 (34.3)	43 (25.4)	597 (21.6)
Number of frail older people participating in the ZWIP, n (%)	61 (85.9)	25 (31.3)	11 (22.4)	55 (47.4)	8 (32.0)	118 (55.4)	12 (27.9)	290 (48.6)
Female, n (%)	34 (55.7)	15 (60.0)	4 (36.8)	40 (72.7)	6 (75.0)	73 (61.9)	10 (83.3)	182 (62.8)
Age, mean (SD)	81.8 (5.4)	81.6 (4.8)	79.2 (5.8)	80.2 (6.2)	82.5 (7.5)	81.1 (5.6)	82.8 (7.5)	81.2 (5.7)
Number of frail older people in the ZWIP who logged on to the ZWIP once, n (%)	9 (14.8)	2 (8.0)	2 (18.2)	8 (14.5)	3 (37.5)	18 (15.3)	1 (8.3)	43 (14.8)
Number of frail older people in the ZWIP who logged on to the ZWIP more than once, n (%)	25 (41.0)	17 (68.0)	5 (45.5)	23 (41.8)	5 (62.5)	36 (30.5)	6 (50.0)	117 (40.3)
Number of professionals participating in the ZWIP, n	31	17	25	43	16	30	16	169^f^
Female, n (%)	21 (67.7)	12 (70.6)	18 (72.0)	33 (76.7)	12 (75.0)	23 (76.7)	13 (81.3)	126 (74.6)
Occupation, n (%)								
General practitioner	6 (19.4)	4 (23.5)	9 (36.0)	8 (18.6)	5 (31.3)	9 (30.0)	3 (18.8)	42 (24.9)
Practice nurse	0 (0.0)	1 (5.9)	1 (4.0)	1 (2.3)	1 (6.3)	6 (20.0)	1 (6.3)	13 (7.7)
District nurse	7 (22.6)	1 (5.9)	3 (12.0)	8 (18.6)	2 (12.5)	0 (0.0)	4 (25.0)	24 (14.2)
Pharmacist	1 (3.2)	1 (5.9)	2 (8.0)	6 (14.0)	2 (12.5)	1 (3.3)	3 (18.8)	15 (8.9)
Physiotherapist	7 (22.6)	4 (23.5)	3 (12.0)	6 (14.0)	3 (18.8)	5 (16.7)	2 (12.5)	30 (17.8)
(Gerontological) social worker	1 (3.2)	2 (11.8)	3 (12.0)	2 (4.7)	0 (0.0)	1 (3.3)	1 (6.3)	9 (5.3)
Hospital-based specialist	1 (3.2)	0 (0.0)	1 (4.0)	2 (4.7)	0 (0.0)	0 (0.0)	0 (0.0)	3 (1.8)
Other	8 (25.8)	4 (23.5)	3 (12.0)	10 (43.3)	3 (18.8)	8 (26.7)	2 (12.5)	33 (19.5)
Number of professionals in the ZWIP of a frail older person, mean (range)	2.5 (0–5)^a^	4.1 (0–8)^b^	1.9 (0–4)	1.8 (0–5)	4.1 (2–6)	2.6 (1–6)^d^	3.0 (1–5)	2.6 (0–8)^g^
Number of professionals in the ZWIP who logged on to the ZWIP once, n (%)	2 (6.5)	1 (5.9)	2 (8.0)	7 (16.7)^c^	2 (12.5)	3 (10.3)^e^	8 (50.0)	25 (15.0)^h^
Number of professionals in the ZWIP who logged on to the ZWIP more than once, n (%)	22 (71.0)	14 (82.4)	20 (80.0)	26 (61.9)^c^	11 (68.8)	23 (79.3)^e^	6 (37.5)	116 (69.5)^h^
Number of messages sent in the ZWIP by professionals, mean (range)	3.6 (0–24)	5.7 (0–46)	0.3 (0–5)	1.3 (0–17)^c^	0.3 (0–3)	0.9 (0–6)^e^	0.7 (0–9)	1.9 (0–46)^h^
Number of messages sent in the ZWIP by frail older people and informal caregivers, mean (range)	1.2 (0–21)	3.2 (0–31)	0.6 (0–2)	0.9 (0–34)	0.1 (0–1)	0.1 (0–4)	0.2 (0–1)	0.8 (0–34)
Number of frail older people in whose ZWIP ≥ 5 messages have been sent, n (%)	7 (11.5)	8 (32.0)	2 (18.2)	3 (5.5)	0 (0.0)	1 (0.8)	0 (0.0)	21 (7.2)

ZWIP = Health and Welfare Information Portal; ^a^n = 46; ^b^n = 24; ^c^n = 42; ^d^n = 117; ^e^n = 29; ^f^as some professionals were involved in the network of more than one general practice, the total number of professionals is less than the sum of professionals in all general practices; ^g^n = 273; ^h^n = 167.

Most interviewees described being involved in the ZWIP of at least some frail older people, varying from one to tens of people. Some used the ZWIP quite often for a limited number of frail older people, whereas others rarely used it. Interviewees described that most frail older people and their informal caregivers made limited use of the ZWIP. On the other hand, they also gave examples of frequent users of the ZWIP. One interviewee described that the use of the ZWIP was limited when the frail older person was in good health, but that its use increased when the frail older person became ill. Table 4 provides some illustrative examples of quotes by interviewees.

**Table 4:** Illustrative quotes of participants

Outcomes of the implementation process	“I have one patient who actually ended up with a ZWIP…and I never hear anything from him” General practitioner1
	“But I think that everyone who participates in the ZWIP here in the municipality…they have asked me to become involved in their network….so it’s tens of people” Other professional1
	“Whereas this week I saw, with another man here in X, he communicates [over the ZWIP] with the general practitioner by himself” Professional conducting the screening1
Appreciation of the implementation strategies	“But those other disciplines, you never or rarely talk to them, and in those three [educational] meetings that we had here it was very interesting to see that, yes, what everyone does, yes, what the added value is of everyone…so you put people in primary care, also due to this project, around the table” Other professional1
	“Yes, it [the educational program] helped, but then it was too lengthy to send all eight general practitioners there” General practitioner2
	“Yes, I really felt [coaching] was very important, for example, starting up a ZWIP account for the first time…just to accompany her one time and to see, and how it is done, yes, that just works better than a paper manual” Professional conducting the screening1
Barriers and facilitators to the ZWIP	“Or older people that say like, yes, I need to call the general practitioner so often, and that is so difficult because he is so difficult to reach, because then I need to tell him my blood sugar for example, and then I have to be on hold and then I finally have the medical assistant, and then there’s an emergency call and I have to wait again. And now I can just type it through a secure system, and then I’m done” Implementation team1
	“I thought it was really good, you [the implementation team] just gave a lot of time and attention, and were very easy to contact and yes, that was very nice, and everyone was really enthusiastic” General practitioner3
	“But the advantage of the ZWIP is of course that it’s a secure network, but that you can choose your own time for responding” Other professional1
	“Yes, I think the application is quite easy to work with” Professional conducting the screening1
	“And in that way I keep thinking like, well, that study has actually come ten years too early… with a generation that’s not, that didn’t grow up with computers, I think that’s a pity” General practitioner1
	“And also, last year it was of course also that issue around the, er, National Electronic Health Record, that made people think like, yes, is everything really that reliable….” Professional conducting the screening2
	“Or the security token didn’t work or they had the wrong token or you know, those actually small things but those were really annoying for the general practitioners” Implementation team1
	“For in these kind of projects, and there’s no way to do it differently, you have parallel development lines, you have simultaneously the trajectories of the educational program that’s being developed, that should start, the information technology, but at a certain time the information technology is not just as ready, and then the information technology is, but the goals or the patient education materials aren’t ready yet. So, and that’s because we were under a lot of pressure with the time…” Implementation team2
	“And then I’m quite a bad one to persuade people [to participate]. Probably because I’m not one-hundred percent convinced myself” Professional conducting the screening3
Recommendations for future implementation of the ZWIP	“You can use it for every disease or every target population…and indeed, also with dementia, in palliative phases of patients when patients are still active themselves…” Implementation team1
	“Yes, those [non-frail older people] who don’t, those who are still quite vital, who do have that age. They would…those are people who are much quicker eh, could work with it before they, before they really…and I think they might be able to benefit from it” Professional conducting the screening1
	“You could stimulate its use in small groups…for once I was in a community centre…then, we just sat with X and some older people, and then really around a round table, just re-enacting it. And then you see that people understand it much quicker and can also see that you are actually communicating” Implementation team3
	“I think that a lot of registration systems that those people have, eh, we have a different kind of registration system and the ZWIP is another, I think that many things can be linked to each other” Professional conducting the screening2

### Factors contributing to the implementation outcomes

#### Exposure to the implementation strategies

All but one of the planned implementation strategies targeting professionals (Table 1) had been available during the implementation period. The development of E-learning for professionals took longer than expected and was therefore not used during the implementation phase. We added one implementation strategy for professionals during the implementation period, i.e. the designation of one key person in each general practice who coordinated the required activities and helped colleagues with questions, as coordinating everything from one central point became too demanding for the implementation team. For frail older people and informal caregivers all planned implementation strategies had been available.

Exposure of professionals and frail older people and informal caregivers to the separate implementation strategies varied over the participating general practices (Table 5). For instance, professionals’ participation in the educational program varied between 60% and 100%. Their overall exposure to coaching was 47%; it was 95% (20 of 21) for professionals conducting the program’s screening for frailty. Of the participating frail older people and informal caregivers, only 62 had used the offered but not obligatory visits by a volunteer to explain the ZWIP, 63% of GPs (19 of 30) had always or often called their frail older patients themselves to ask them to participate in the screening.

**Table 5:** Exposure of professionals, frail older people and informal caregivers to the program’s implementation strategies

	**General practice 1**	**General practice 2**	**General practice 3**	**General practice 4**	**General practice 5**	**General practice 6**	**General practice 7**	**Total**
**Exposure of professionals**	n = 17	n = 13	n = 16	n = 18	n = 12	n = 20	n = 12	n = 115^n^
Involvement of professionals in development								
Aware of involvement, yes, n (%)	15 (88.2)	10 (76.9)	10 (71.4)^e^	16 (88.9)	8 (66.7)	12 (60.0)	9 (75.0)	84 (75.0)^o^
Involved, yes, n (%)	3 (17.6)	3 (23.1)	3 (20.0)^f^	3 (16.7)	0 (0.0)^a^	1 (5.0)	1 (8.3)	15 (13.4)^o^
Flyers								
Received, yes, n (%)	11 (64.7)	6 (46.2)	9 (60.0)^f^	14 (77.8)	8 (66.7)	9 (47.4)^k^	5 (41.7)	63 (56.3)^o^
Read, yes, n (%)	11 (100.0)^a^	6 (100.0)^c^	9 (100.0)^g^	10 (76.9)^h^	8 (100.0)^j^	9 (100.0)^g^	5 (100.0)^m^	59 (95.2)^p^
Educational program								
Participated, yes, n (%)	13 (76.5)	12 (92.3)	9 (60.0)^f^	14 (77.8)	11 (91.7)	19 (95.0)	12 (100.0)	90 (79.6)^q^
Coaching for professionals conducting screening								
Received, yes, n (%)	8 (47.1)	5 (38.5)	11 (68.8)	10 (55.6)	3 (25.0)	12 (60.0)	4 (33.3)	54 (47.0)
Telephonic helpdesk								
Contacted, yes, n (%)	11 (64.7)	5 (38.5)	9 (56.3)	11 (61.1)	3 (25.0)	9 (45.0)	3 (25.0)	53 (46.1)
Newsletter								
Received, yes, n (%)	10 (58.8)	13 (100.0)	11 (68.8)	12 (66.7)	7 (63.6)^a^	13 (72.2)^l^	7 (58.3)	74 (66.1)^o^
Financial compensation								
Aware of receiving it when applicable, yes, n (%)	12 (75.0)^b^	12 (100.0)^d^	8 (61.5)^h^	12 (70.6)^i^	9 (75.0)	11 (68.8)^b^	12 (100.0)	77 (73.3)^r^
**Exposure of frail older people and informal caregivers**	n = 61	n = 25	n = 11	n = 55	n = 8	n = 118	n = 12	n = 290
Visit or call by a volunteer who explains the ZWIP								
Number of older people or informal caregivers visited by volunteer, n	5	8	1	1	2	28	1	62^s^
Telephonic helpdesk								
Number of contacts of helpdesk with frail older people or informal caregivers, n	16	8	1	9	2	12	3	54^t^

ZWIP = Health and Welfare Information Portal; ^a^n = 11; ^b^n = 16; ^c^n = 6; ^d^n = 12; ^e^n = 14; ^f^n = 15; ^g^n = 9; ^h^n = 13; ^i^n = 17; ^j^n = 8; ^k^n = 19; ^l^n = 18; ^m^n = 5; ^n^n = 115 due to unknown general practice for 7 professionals; ^o^n = 112; ^p^n = 62; ^q^n = 113; ^r^n = 105; ^s^n = 62 due to unknown general practice for 16 visits; ^t^n = 54 due to unknown general practice for 3 calls

#### Appreciation of the implementation strategies

Of the surveyed professionals who had participated in the educational program, 70% (63 of 89) considered it (very) necessary for being able to work with the ZWIP. Interviewees confirmed this, as they felt that meeting each other and gaining knowledge about each others’ expertise during the educational program facilitated collaboration, and they appreciated the opportunity to ask questions and to practice working with the ZWIP. However, they did feel that the educational program could have been shorter, and that too much time had elapsed between the educational program and the first frail older persons having a ZWIP. As for the coaching, only 26% (14 of 53) of surveyed professionals who had received coaching felt they would not be able to work with the ZWIP without it. However, interviewees did consider coaching necessary, as they appreciated the assistance of someone experienced in conducting the screening and entering data in the ZWIP. The helpdesk was considered (very) necessary by 77% (41 of 53) of surveyed professionals who had contacted it, and interviewees agreed that it was useful (Tables 4 and 6).

**Table 6:** Perceived value of the implementation strategies used

**Implementation strategy**	**n = 115**^**a**^
Involvement of professionals in development	
Involvement of GPs/professionals important; yes, n (%)	95 (86.4)^b^
Flyers	
Flyers important for deciding about participation, yes, n (%)	35 (59.3)^c^
Estimated relevance to future users on a scale of 1–10, mean (SD)	6.8 (1.6)^d^
Educational program	
Necessary for being able to work with ZWIP; yes, n (%)	63 (70.8)^e^
Estimated relevance to future users on a scale of 1–10, mean (SD)	7.5 (1.6)^f^
Coaching	
Able to work with ZWIP without coaching; no, n (%)	14 (26.4)^g^
Estimated relevance to future users on a scale of 1–10, mean (SD)	6.9 (1.7)^h^
E-coaching	
Necessary for being able to work with ZWIP; yes, n (%)	8 (38.1)^i^
Estimated relevance to future users on a scale of 1–10, mean (SD)	6.7 (1.6)^j^
Telephonic helpdesk	
Necessary for being able to work with ZWIP; yes, n (%)	41 (77.4)^g^
Estimated relevance to future users on a scale of 1–10, mean (SD)	7.3 (1.6)^f^
Newsletter	
Newsletter important for staying up-to-date about the program; yes, n (%)	35 (46.7)^k^
Estimated relevance to future users on a scale of 1–10, mean (SD)	5.9 (1.5)^d^
Financial compensation	
Financial compensation necessary for future professionals; yes, n (%)	27 (56.3)^l^
Estimated relevance to future users on a scale of 1–10, mean (SD)	6.7 (2.0)^h^
Possibility to adapt the ZWIP to meet local circumstances	
Estimated relevance to future users on a scale of 1–10, mean (SD)	8.0 (1.4)^f^

ZWIP = Health and Welfare Information Portal; ^a^Professionals could answer affirmatively, neutral or negatively, n varies as questions concerning necessity and importance were only answered by professionals exposed to the implementation strategy; ^b^n = 110; ^c^n = 59; ^d^n = 106; ^e^n = 89; ^f^n = 107; ^g^n = 53; ^h^n = 105; ^i^n = 21; ^j^n = 104; ^k^n = 75; ^l^n = 48.

#### Barriers and facilitators

Interviewees stated that for all target populations experiencing problems with interprofessional communication or contacting professionals had been an important incentive for participating in the ZWIP. Additional facilitators for professionals were appreciating that the ZWIP could be used at a time of their choosing, sympathizing with the idea of the ZWIP, enjoying participating in something new, the ZWIP application being user-friendly and receiving sufficient support in working with the ZWIP. Additional facilitators for frail older people were wanting to keep control of their own care, appreciating that their message in the ZWIP was directly and quickly answered by their own GP instead of by the medical assistant, participation of an informal caregiver and the GP being involved.

On the other hand, preferring to have face-to-face contact presented an important barrier to the use of the ZWIP for all target populations. Barriers specific for professionals were considering the ZWIP too early for the current generation of older people whose computer-literacy is limited and having doubts about whether the ZWIP is the best way to improve care. In addition, interviewees considered time constraints an important barrier, even though 67% (64 of 96) of surveyed professionals considered the time spent on using the ZWIP (very) limited. Further, limited use of the ZWIP by both professionals and frail older people presented a barrier to respectively 52% (50 of 94) and 60% (56 of 94) of surveyed professionals (Table 7). Interviewees agreed with this, as they considered not being invited into the ZWIP of frail older people much, receiving few messages, and not all professionals in their work area being familiar with the ZWIP an important barrier for its use. A final barrier described were the start-up problems experienced by professionals, which included the ZWIP application not working correctly, lack of clarity about the eligibility criteria for older people, and receiving financial compensation too late. Interviewees felt that at the start of the project, the implementation team had struggled with translating the ideas behind ZWIP into everyday practice, sometimes causing support to be lacking or too late. Interviewed members of the implementation team acknowledged these start-up problems, and explained the problems with the ZWIP application by the limited time available for its initial development, resulting in improvements of the ZWIP continuing alongside its implementation. Further, interviewees of the implementation team described that the need for local professionals and organizations to get ready to work together first, and the obligated but time-consuming population-based screening had slowed down the implementation of the ZWIP. Barriers specific for frail older people included considering the ZWIP not useful or quite a fuss, and considering the ZWIP something for professionals. In addition, frail older people were not always invited to participate by a motivated professional or were not considered eligible to participate by professionals. However, the main barriers for frail older people related to computers, i.e. not having a computer, not being comfortable with or capable of working with a computer, concerns about the security of the ZWIP and not yet being familiar with the ZWIP. Although we did direct several implementation strategies at these barriers, such as asking an informal caregiver to use the ZWIP for the frail older person and offering a visit by a volunteer to explain the ZWIP, one interviewee remarked that sometimes, although explicitly offered, frail older people did not want to use these strategies as they did not want to burden them or did not want yet another unknown person in their house (Table 4).

**Table 7:** Experienced barriers and facilitators to working with the ZWIP

**n = 105**	**Disagree, n (%)**	**Neutral, n (%)**	**Agree, n (%)**
The data included in the ZWIP are sufficiently safeguarded	2 (2.0)^a^	48 (49.0)^a^	48 (49.0)^a^
The data included in the ZWIP are accurate	5 (5.1)^b^	45 (45.5)^b^	49 (49.5)^b^
The data included in the ZWIP are not up-to-date	32 (32.3)^b^	54 (54.5)^b^	13 (13.1)^b^
The data included in the ZWIP provide me insufficient information	30 (30.3)^b^	51 (51.5)^b^	18 (18.2)^b^
The data included in the ZWIP are way too extensive	38 (38.0)^c^	61 (61.0)^c^	1 (1.0)^c^
I feel that the ZWIP is very user-friendly	11 (11.1)^b^	42 (42.4)^b^	46 (46.5)^b^
I feel that working with computers is uncomfortable	81 (81.8)^b^	11 (11.1)^b^	7 (7.1)^b^
I feel that the instruction during the educational program was sufficient to be able to work with the ZWIP	6 (6.3)^d^	17 (17.7)^d^	73 (76.0)^d^
Working with the ZWIP is too complicated	45 (46.9)^d^	38 (39.6)^d^	13 (13.5)^d^
Working with the ZWIP ultimately saved me time	46 (48.9)^e^	42 (44.7)^e^	6 (6.4)^e^
I feel that the ZWIP does not fit into my regular working pattern	68 (68.7)^b^	20 (20.2)^b^	11 (11.1)^b^
Working with the ZWIP gives me the enough leeway to incorporate the goals of the frail older person in decisions about his/her care	10 (10.9)^f^	41 (44.6)^f^	41 (44.6)^f^
Working with the ZWIP is difficult since other professionals involved do not use the ZWIP much	8 (8.5)^e^	36 (38.5)^e^	50 (52.1)^e^
Working with the ZWIP is difficult since the frail older person and informal caregiver do not use the ZWIP much	7 (7.4)^e^	31 (33.0)^e^	56 (59.6)^e^

ZWIP = Health and Welfare Information Portal; Total may not amount to 100.0% due to rounding; ^a^n = 98; ^b^n = 99; ^c^n = 100; ^d^n = 96; ^e^n = 94; ^f^n = 92.

### Improving a future implementation of the ZWIP

Interviewees made several recommendations for improving future implementations of the ZWIP. These included shortening the educational program, having an e-learning and a website available and using early adopters as advocates for the program. For frail older people, interviewees suggested the use of an additional implementation strategy, i.e. organising meetings for all frail older people in which they can learn about the ZWIP and practice its use, in order to familiarise them with the ZWIP within a more comfortable context. Interviewees considered the ZWIP useful for other populations as well, e.g. for frail people younger than 70 years, non-frail older people, psychiatric patients, palliative patients, patients with diabetes or COPD and for problematic family situations. Last, interviewees shared some considerations for improvements of the ZWIP itself, which included linking the ZWIP to their own electronic health records, and enabling professionals to use the ZWIP more flexibly, i.e. to use only those parts needed (Table 4).

## Discussion

This study describes the implementation of the ZWIP. By the end of the implementation period, 290 frail older people and 169 professionals were involved in the ZWIP. Their use of the ZWIP varied. The implementation strategies were generally delivered as planned for professionals. However, the exposure of frail older people and informal caregivers to some of the implementations strategies, such as their use of the optional instruction about the ZWIP application by volunteers was less than intended. Professionals were generally positive about the implementation process, especially about the interprofessional educational program and the helpdesk. Factors that facilitated the implementation of the ZWIP were frail older people and professionals feeling the need to enhance interprofessional collaboration and the ZWIP application being user-friendly, barriers were the low computer-literacy of frail older people, start-up problems, a preference for personal contact and limited use of the ZWIP by others. Interviewees recommended adapting the implementation strategies to make them more efficient, for example by shortening the educational programme, to use the ZWIP for other target populations as well, and to add new strategies that may help frail older people to feel more comfortable with computers and the ZWIP.

### Outcome of the implementation process

Overall, the results of the implementation were positive. First, the frail older people who were the target population of the ZWIP are likely to be among the most difficult populations to engage in an e-health intervention, as they feel less comfortable and competent with computers than younger populations [19,20]. Therefore, the recruitment of 290 frail older participants (49% of those invited to participate), who were not previously selected for having computer skills, is quite a positive outcome. Of course, their actual use of the ZWIP during the implementation period varied, but those who never or rarely used the ZWIP may not have had a reason to use it, as all went well. In addition, for several frail older people a ZWIP was created near the end of the implementation period, which resulted in them having had limited time to use it. However, participants could continue to use the ZWIP for one year following the end of the implementation period. Additional positive results of the implementation are that the interviewed professionals recommended using the ZWIP for other target populations recommended also using the ZWIP for other target populations, and that professionals and frail older people not yet involved in the current research project had approached us to ask whether they could use the ZWIP as well.

Important factors that contributed to these outcomes were the involvement of future target populations throughout the development process; the implementation strategies such as the interprofessional educational program and the helpdesk, which were considered particularly useful by professionals; and the widely acknowledged need to improve the care for the growing number of frail older people and to improve the communication and collaboration among professionals [21,22]. As the ZWIP incorporates many components of the currently advocated introduction of the patient-centred medical home [23], e.g. patient-centeredness, care-coordination and the use of e-health such as shared Electronic Health Records to improve quality of care [23], it fits very well in the improvements currently recommended for primary care.

### Comparison to the literature

To our knowledge, the ZWIP is one of the first e-health interventions to combine a multidisciplinary shared Electronic Health Record with interprofessional and patient-professional communication. This, added to the limited number of publications concerning the results of the implementation of e-health interventions [12], makes comparisons to other studies difficult. However, there have been several articles published concerning the barriers for the implementation of e-health interventions such as electronic medical records or electronic communication. These largely agree with the barriers found in this study, i.e. preferring personal contact, being worried about security of data and time constraints [13,24-26], even though the results on this latter barrier were somewhat mixed in our study, with about two-third of professionals reporting that they considered the time spent on using the ZWIP limited, whereas interviewees reported that time constraints did present a barrier to using the ZWIP. Additional barriers specific to this study were the low computer-literacy of frail older people and the experienced start-up problems.

### Strengths and limitations

Our study had some limitations. First, as a result of a deliberate choice, frail older people and informal caregivers’ experiences with the implementation process were only evaluated indirectly. We do acknowledge the limitations of receiving such indirect information. However, we expect that we have been able to give an overall impression of their experiences with the projects’ implementation. Second, the implementation was evaluated by members of the project team who were involved in its implementation. Although we used objective quantitative data sources, and ensured that the interviews with primary care professionals were conducted by an objective research assistant, this may have affected our results. Third, the study was conducted in the Netherlands, which healthcare system is characterized by each patient having their own GP, usually over an extended time period. This may limit the generalisability of our findings to healthcare systems which do not have such a strong primary care foundation. On the other hand, the study had several important strengths, which include the large number of participants, including a large number of frail older people who are usually more difficult to recruit for such projects and a large group of professionals of whom a large majority (87%) had not been involved in the development process themselves, the use of a mixed-methods design which combined multiple data sources, and the ZWIP being implemented directly in everyday practice for use in regular care.

## Conclusions

This study has described the implementation of an innovative e-health intervention for community-dwelling frail older people, informal caregivers and primary care professionals, which had positive results. The implementation strategies for professionals, especially the involvement of the target populations in its development, the educational program which resulted in professionals getting to know each other personally and the helpdesk, as well as the experienced need for improving care for frail older people contributed to this. However, the strategies intended to improve frail older people’s computer literacy did not always succeed as exposure to these strategies was limited. Therefore, both additional strategies targeting frail older people’s computer literacy and reviewing the ZWIP to optimise its user-friendliness are needed. Nevertheless, as e-health is an important medium for overcoming healthcare fragmentation and facilitating patient involvement, but its adoption in everyday practice remains a challenge, the results of this implementation are promising.

### Practice implications

Based on the current study, several recommendations can be made for the implementation of comparable e-health interventions. First, when e-health innovations are directed at populations who currently have limited computer literacy, such as frail older people, implementation efforts should focus on improving this by e.g. a comprehensive training program [20]. Piloting the implementation strategies selected for this aim, to ensure that they are able to meet the needs of the target population, is highly recommendable. Second, as a preference for personal contact continues to be an important barrier for the use of e-health by both patients and professionals, it should be addressed during the implementation, e.g. by emphasizing that e-health is meant to be an addition to and not a replacement of the existing spectrum of communication methods and by providing professionals engaging in the use of electronic communication the opportunity to get acquainted with each other during the implementation. Last, although some ongoing development is probably unavoidable with many innovative e-health interventions, the resulting inconvenience for professionals should be restricted to a minimum, as the start-up problems caused by working with an application under development are likely to deter participants who were hesitant to adopt these techniques to begin with.

## Competing interests

The authors declare that they have no competing interests.

## Authors’ contributions

SR was involved in the design of the study, conducted the interviews, conducted the data analysis and drafted the manuscript; MP was involved in the design of the study, conducted the interviews, conducted the data analysis and critically reviewed the manuscript; MH was involved in the design of the study and critically reviewed the manuscript; LvN conducted the interviews and critically reviewed the manuscript; HS was involved in the design of the study and critically reviewed the manuscript; CvW critically reviewed the manuscript; MOR critically reviewed the manuscript; TvA was involved in the design of the study and critically reviewed the manuscript; MMH was involved on the design of the study and critically reviewed the manuscript; RM was involved in the design of the study and critically reviewed the manuscript. All authors read and approved the final manuscript.

## Pre-publication history

The pre-publication history for this paper can be accessed here:

http://www.biomedcentral.com/1472-6963/12/251/prepub

## Supplementary Material

Additional file 1 **Movie demonstrating the use of the Health and Welfare Information Portal by a frail older person and her informal caregiver.** (WMV 16069 kb) Click here for file
